# Polymodal Responses in *C. elegans* Phasmid Neurons Rely on Multiple Intracellular and Intercellular Signaling Pathways

**DOI:** 10.1038/srep42295

**Published:** 2017-02-14

**Authors:** Wenjuan Zou, Hankui Cheng, Shitian Li, Xiaomin Yue, Yadan Xue, Sixi Chen, Lijun Kang

**Affiliations:** 1 Department of Neurobiology, Institute of Neuroscience, Key Laboratory of Medical Neurobiology of the Ministry of Health of China, Zhejiang University School of Medicine, Hangzhou, China; 2 School of Physics and Information Engineering, Fuzhou University, Fuzhou, Fujian, China

## Abstract

Animals utilize specialized sensory neurons enabling the detection of a wide range of environmental stimuli from the presence of toxic chemicals to that of touch. However, how these neurons discriminate between different kinds of stimuli remains poorly understood. By combining *in vivo* calcium imaging and molecular genetic manipulation, here we investigate the response patterns and the underlying mechanisms of the *C. elegans* phasmid neurons PHA/PHB to a variety of sensory stimuli. Our observations demonstrate that PHA/PHB neurons are polymodal sensory neurons which sense harmful chemicals, hyperosmotic solutions and mechanical stimulation. A repulsive concentration of IAA induces calcium elevations in PHA/PHB and both OSM-9 and TAX-4 are essential for IAA-sensing in PHA/PHB. Nevertheless, the PHA/PHB neurons are inhibited by copper and post-synaptically activated by copper removal. Neuropeptide is likely involved in copper removal-induced calcium elevations in PHA/PHB. Furthermore, mechanical stimulation activates PHA/PHB in an OSM-9-dependent manner. Our work demonstrates how PHA/PHB neurons respond to multiple environmental stimuli and lays a foundation for the further understanding of the mechanisms of polymodal signaling, such as nociception, in more complex organisms.

Animals employ sensory neurons to detect external stimuli such as the presence of chemicals or aspects of temperature or touch. These specialized neurons are often polymodal where a single type of sensory neuron may respond to a number of different kinds of stimuli^1^. Polymodal nociceptive neurons in the mammalian skin, for example, have the ability to respond to heat, noxious chemicals and mechanical stimuli^2^. How these neurons discriminate between these different kinds of stimuli remains largely unknown.

Simple organisms such as *C. elegans* provide useful platforms to tease out how signaling molecules and neuronal circuits generate complex behaviors. *C. elegans* is equipped with a small nervous system consisting of 302 neurons. Many of these neurons may be multifunctional^3^,^4^. 32 presumed chemosensory neurons in the amphid, phasmid and inner labial organs are either directly or indirectly exposed to the environment^5^. The amphids in the head are the largest sensory organs in *C. elegans*. They consist of 12 pairs of sensory neurons capable of sensing numerous sensory stimuli^1^,^5^. The phasmids are bilateral sensory organs located in the tail of the worm. They contain similar structures to those of the amphids^3^,^6^. A previous study has reported that the antagonistic activity of the amphid neurons, mainly ASH, and the phasmid neurons, PHA and PHB, integrate to generate avoidance behaviors^6^. In Hilliard’s study, the PHA/PHB neurons were suggested to probably act as the initial chemo-sensors in detecting detergent (SDS) where the decision to initiate avoidance behavior were considered to also incorporate information from the ASH neurons. In this way the worms were enabled to avoid or escape from noxious SDS stimulus^6^,^7^. Ablation of the PHA/PHB neurons also caused significant deficiencies in the avoidance responses to harsh touch. This indicates that these neurons also play a specific role in the noxious touch sensation^8^. However, the nature of such responses of phasmid neurons to environmental stimuli has yet to be determined on a cellular level and the underlying molecular mechanisms remain unclear.

In this study, we show that the PHA/PHB neurons respond to a wide range of aversive stimuli including aversive odors, copper, alkaline solution, hyperosmotic solution, and harsh touch. We further identify critical roles for the TRPV protein OSM-9, the CNG channel protein TAX-4 and the post-synaptic neuropeptide in the sensory transduction of PHA/PHB. Our data suggests that the PHA/PHB neurons are polymodal neurons employing an elaborate combination of intracellular and intercellular signaling pathways to detect and process environmental stimuli.

## Results

### The PHA/PHB neurons respond to a wide range of aversive stimuli

**Figure 1:**
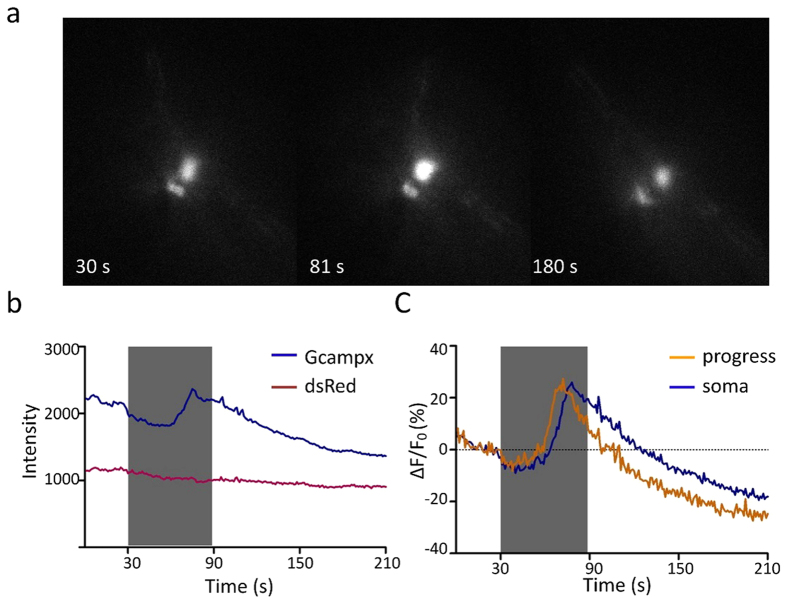
SDS induces calcium elevations in the PHA/PHB neurons. (**a**) Calcium transients in the PHA/PHB neurons visualized with GCaMP5.0. A red fluorescent protein DsRED was co-expressed as a reference. Individual frames taken before, during and after application of 1:100 SDS are shown. (**b**) Fluorescence intensities reflect an increase in the calcium intracellular level in PHA/PHB neurons. (**c**) Calcium transients in the cell body and dendrites of PHA/B have similar profiles in response to a 60 s stimulus of 1:100 SDS.

To monitor the activities of the PHA/PHB neurons, we generated a transgenic strain in which the calcium indicator protein GCaMP5.0 was transcribed under the control of the *ocr-2* promoter^9^. Previous studies have reported that PHA/PHB neurons are required for detergent (SDS)-evoked avoidance behavior^6^,^7^. Consistent with these studies, we observed reliable calcium elevations in both the soma and the processes of the PHA/PHB neurons upon perfusion of 1% SDS to the tail of the worm (Fig. 1a–c).

**Figure 2:**
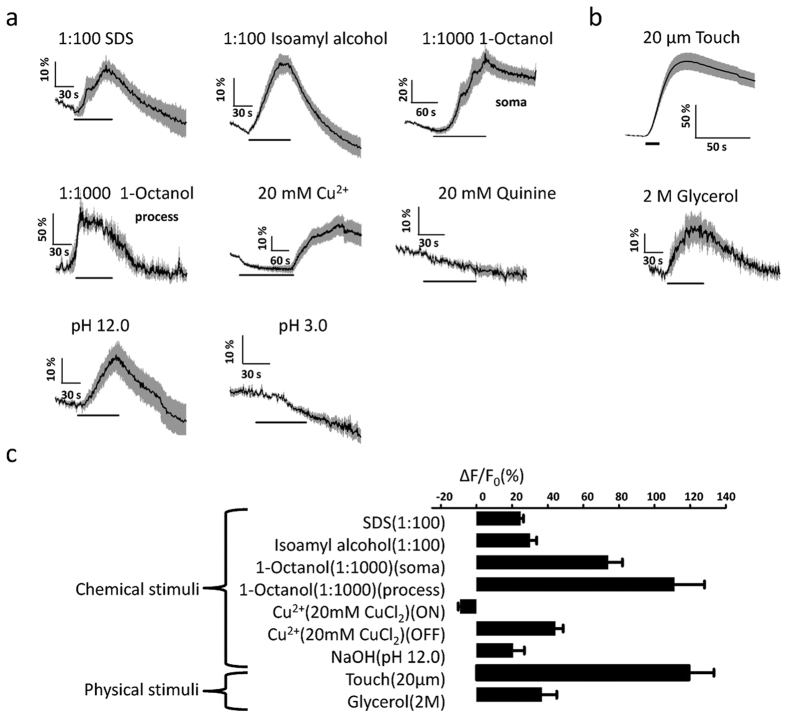
Noxious stimuli triggered calcium transients in the PHA/PHB neurons. (**a**) A variety of noxious chemical stimuli triggered calcium transients in the PHA/PHB neurons. The solid line shows the average fluorescence change and the shading around it indicate the ± SEM. Horizontal lines indicate the duration of application for each stimulus. Notably, 20 mM Cu^2+^ induced a decrease of calcium level in the PHA/PHB neurons. For 1-Ocanol induced-calcium elevations, both the soma and the progress were shown. (**b**) Calcium transients in the PHA/PHB neurons in response to physical stimuli. (**c**) Maximal calcium changes of the PHA/PHB neurons to the application of noxious stimuli. N > = 6 for each experiment, Data are mean ± SEM.

We then sought to discover whether the PHA/PHB neurons could be activated by other chemical and physical stimuli. We observed robust calcium transients in the PHA/PHB neurons during the stimulation of the worms with aversive odors such as isoamyl alcohola (1:100 IAA) and 1-octanol (1:1000) and an alkaline solution of pH 12. Harsh touch (20 μm displacement) and hyperosmotic solution (2 M glycerol) also induced robust calcium transients in the PHA/PHB neurons. However, no such response was observed with the perfusion of the bath solution, alkaloid quinine (20 mM), or an acidic solution of pH 3 (Fig. 2a and b). Interestingly, the calcium levels of the PHA/PHB neurons decreased upon the application of copper heavy metal ions and were increased by copper removal (Fig. 2a,b). No detectable calcium variation was observed with the application of attractive odorants such as butanone. Notably, we did not observed any differences between the responses of PHA and PHB to these stimuli. These observations suggest that PHA/PHB are polymodal neurons responding to noxious chemical and physical stimuli.

### PHA/PHB neurons function as primary sensory neurons for sensing odorants

**Figure 3:**
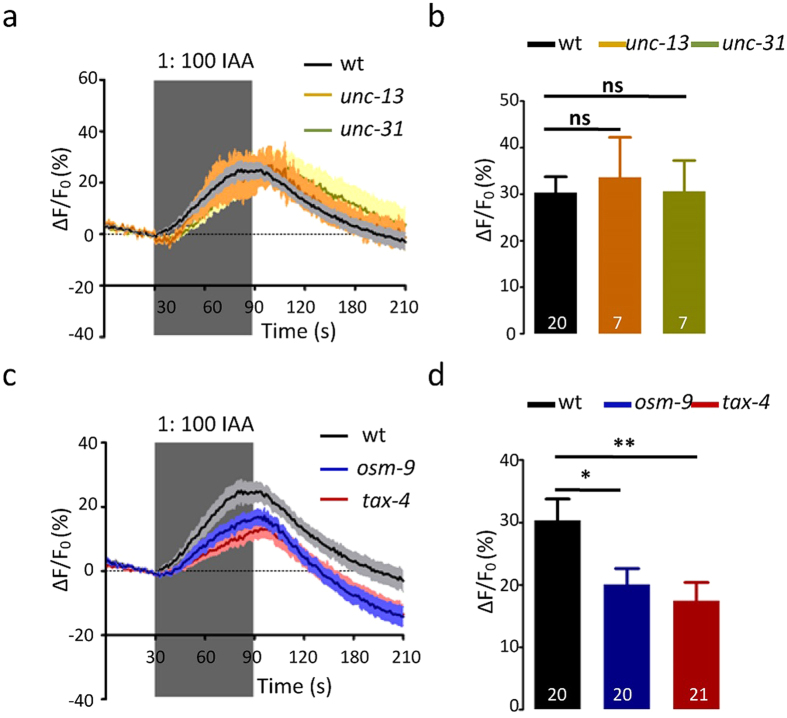
The PHA/PHB neuronscell-autonomously respond to IAA. (**a,b**) 1:100IAA induced-calcium transients in the PHA/PHB neurons in wild-type worms, *unc-13* mutants and *unc-31* mutants. (**a**) Fluorescence changes (mean ± SEM); (**b**) Bar graphs. (**c,d**) 1:100IAA induced-calcium transients in the PHA/PHB neurons in wild-type worms, *osm-9* mutants and *tax-4* mutants. (**c**) Fluorescence changes (mean ± SEM); (**d**) Bar graphs. Error bars indicate mean ± S.E.M.; ns, not significant, P > 0.05; *P < 0.05; **P < 0.01; two-tailed paired t-test.

One possibility is that calcium elevations in the PHA/PHB neurons upon exposure to sensory stimuli occur post-synaptically and are induced by other neurons. Therefore, we tested the IAA-induce responses in PHA/PHB in *unc-13* mutant worms and *unc-31* mutant worms. In this *unc-13* encodes the ortholog of the mammalian Munc13 which is required for neurotransmitter release from synaptic vesicle^10^,^11^ and *unc-31* encodes the ortholog of the mammalian CAPS proteins and is essential for neuropeptide release from dense core vesicles (DCVs)^10^,^11^. Notably, IAA-induced calcium elevations in PHA/PHB in *unc-13* and *unc-31* background were similar to those of wild-type worms. This seems to confirm that PHA and PHB are the primary sensory neurons for sensing IAA (Fig. 3a,b).

### IAA-sensing of the PHA/PHB neurons is dependent in TAX-4 and OSM-9

We then investigated the molecular mechanisms of IAA-sensing in PHA/PHB. TAX-4, a subunit of a cyclic nucleotide gated channel involved in chemotaxis mediated by the AWC neurons, has been implicated as required for PHA/PHB-mediated avoidance response to SDS^6^,^12^. We found that IAA-induce responses in PHA/PHB were dramatically diminished in *tax-4* mutant worms (Fig. 3c,d). Sensory transduction in the ASH neurons in response to noxious osmotic shock, heavy metal ions and volatile chemical and alkaline solutions have all been noted to be mediated by OSM-9, a TRPV-related cation channel^1^,^13^. OSM-9 is expressed in PHA/PHB as well as in some amphid sensory neurons such ASH and AWA^14^. Interestingly, IAA-induced responses in PHA/PHB were also significantly weaker in *osm-9* mutants than in wild-type worms (Fig. 3c,d). This demonstrates that both TAX-4 and OSM-9 are required for IAA-sensing in the PHA/PHB neurons.

### Copper inhibits the PHA/PHB neurons

**Figure 4:**
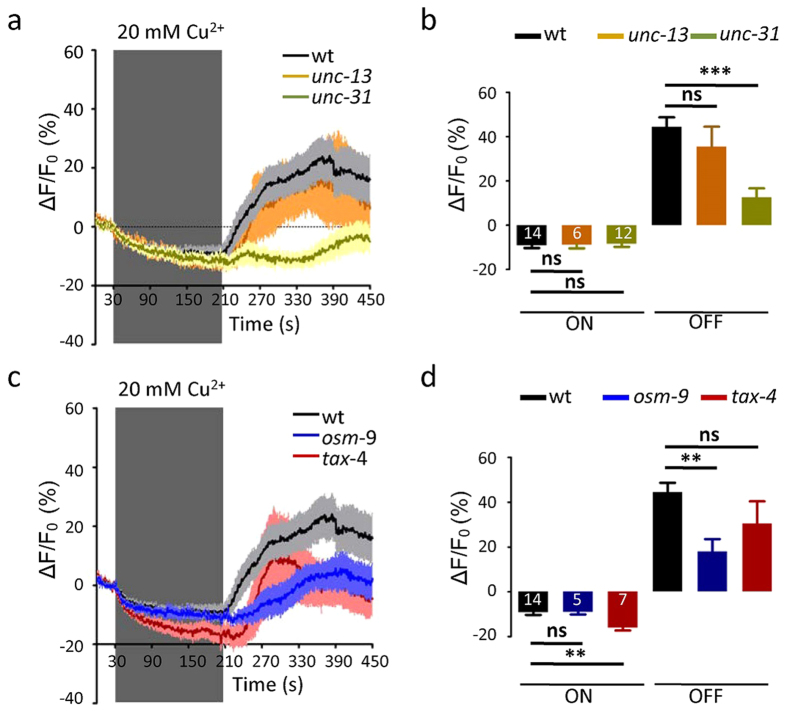
Copper-induced calcium variations in the PHA/PHB neurons. (**a,b**) Cu^2+^ (20 mM) induced-calcium variations in the PHA/PHB neurons in wild-type worms, *unc-13* mutants and *unc-31* mutants. (**a**) Fluorescence changes (mean ± SEM); (**b**) Bar graphs. (**c,d**) Cu^2+^ (20 mM) induced-calcium variations in the PHA/PHB neurons in wild-type worms, *osm-9* mutants and *tax-4* mutants. (**c**) Fluorescence changes (mean ± SEM); (**d**) Bar graphs. Error bars indicate mean ± S.E.M.; ns, not significant, P > 0.05; **P < 0.01; ***P < 0.001; two-tailed paired Student t-test.

Both IAA and copper activates ASH neurons^1^,^15^. Unexpectedly, we found that the calcium levels in the PHA/PHB neurons were decreased by the application of copper (an “ON” response), and were increased by copper removal (an “OFF” response) (Fig. 4a,4b). Neither the “ON” response nor the “OFF” response was affected by the loss of UNC-13 (Fig. 4a,b). However, the “OFF” response was abolished in *unc-31* mutant worms (Fig. 4a,b). These results indicate that copper autonomously inhibits PHA/PHB at a cellular level. Meanwhile, the PHA/PHB neurons may be post-synaptically activated by copper removal via neuropeptides. The Cu^2+^ -induced “ON” response in PHA/PHB was diminished in *osm-9* mutant worms. Nevertheless, TAX-4 was required for “OFF” responses (Fig. 4c,d). These data suggests that copper inhibits PHA/PHB in an OSM-9-dependent manner, and both TAX-4 and neuropeptides are involved in copper removal-induced calcium elevations in PHA/PHB.

### PHA/PHB neurons sense mechanical stimulation in an OSM-9-dependent manner

**Figure 5:**
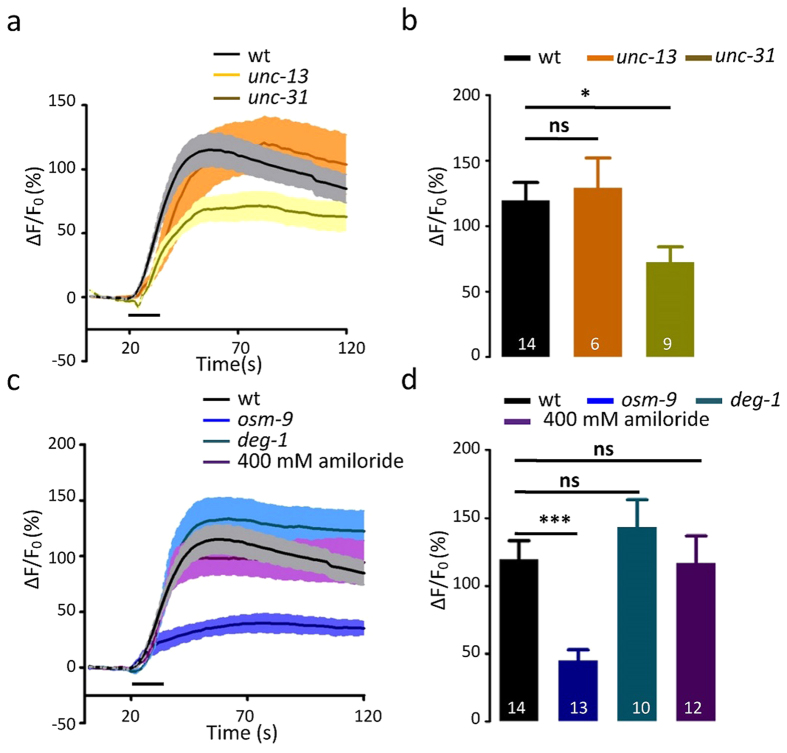
Touch-evoked calcium transients in the PHA/PHB neurons. (**a,b**) Touch induced-calcium transients in the PHA/PHB neurons in wild-type worms, *unc-13* mutants and *unc-31* mutants. (**a**) Fluorescence changes (mean ± SEM); (**b**) Bar graphs. (**c,d**) Touch induced-calcium transients in the PHA/PHB neurons in wild-type worms, wild-type worms treated with amiloride, *osm-9* mutants, and *deg-1* mutants. (**c**) Fluorescence changes (mean ± SEM); (**d**) Bar graphs. Error bars indicate mean ± S.E.M.; ns, not significant, P > 0.05; *P < 0.05; ***P < 0.001; two-tailed paired t-test.

Laser ablation of the PHA/PHB neurons reduces response to harsh touch. This shows that these neurons are also involved in mechano-sensation^8^. Consistent with the behavioral phenotype, we observed robust touch-induced calcium elevations in PHA/PHB (Fig. 5a,b). Touch-induced calcium elevations in PHA/PHB were not reduced in *unc-13* mutant worms and were only slightly smaller in *unc-31* mutant worms than those in the wild-type, which indicates that PHA and PHB are likely mechano-receptor cells (Fig. 5a,b).

Three mechano-gated channels have been identified in *C. elegans*, the two amiloride-sensitive sodium channel (ENaC) proteins MEC-4 and DEG-1, and the TRPN (nomPC) protein TRP-4^16^,^17^,^18^. Since MEC-4 and TRP-4 are not expressed in the PHA/PHB neurons^17^,^18^,^19^, we examined touch-induced response in PHA/PHB in *deg-1* mutant worms. We found that the touch-induced calcium elevations in the PHA/PHB neurons were normal in *deg-1* mutant worms (Fig. 5c,d). Furthermore, the ENaC blocker amiloride failed to affect the touch-induced calcium elevations in PHA/PHB (Fig. 5c,d). This demonstrates that ENaC channels are not involved in mechano-transduction in PHA/PHB. OSM-9 is required for touch-evoked responses in the ASH neurons^1^. We found touch-induced calcium elevations in PHA/PHB were dramatically reduced in o*sm-9* mutant worms, indicating that OSM-9 plays a role in mediating PHA/PHB excitation in response to mechanical stimulation.

## Discussion

In this study, we demonstrate that the *C. elegans* phasmid neurons PHA and PHB are polymodal sensory neurons responding to harmful chemicals and mechanical stimulation. We show that the TRPV channel OSM-9 is essential for both IAA-sensation and touch-sensation, but not for copper-induced calcium variations in PHA/PHB. The CNG channel TAX-4 is especially required for chemo-sensation in these neurons. In addition, neuropeptides are likely required for the copper removal induced-calcium elevations in PHA/PHB.

In *C. elegans*, two GPCR-related signal transduction systems are prominent in chemo-sensation. One relies upon CNG channel and another is mediated by TRPV channels^5^,^15^. In the AWC neurons, odorants bind to the GPCR receptor and activate Gα proteins. This leads to drop of the intracellular level of cGMP, thereby closing the CNG channels TAX-2/TAX-4 and hyperpolarizing the cell^5^,^20^. The CNG channels also mediate thermo-sensation in the AFD neurons and photo-sensation in the ASJ neurons^21^,^22^. The TRPV channel OSM-9 has been proposed to mediate depolarization following all chemical stimuli sensed by the ASH neurons^1^,^13^. Interestingly, here we found both TAX-4 and OSM-9 were essential for IAA-sensation in the PHA/PHB neurons, while OSM-9 and TAX-4 were involved in the copper-induced “ON” and “OFF” responses, respectively, in PHA/PHB. These observations suggest that the PHA/PHB neurons represent distinct mechanisms of chemo-transduction from the amphid sensory neurons. Additionally, we found that copper removal post-synaptically activated the PHA/PHB neurons via neuropeptides. This indicates that the activities of PHA/PHB can also be modulated by other neurons.

Two classes of mechano-gated channels have been identified in *C. elegans*. The first is the amiloride-sensitive ENaC channel subfamily and includes MEC-4 and DEG-1. The second is the TRP subfamily which includes TRP-4^16^,^17^,^18^. MEC-4 is expressed in the six touch receptor neurons including ALM, AVM, PLM, and PVM. MEC-4 may form a heteromeric mechano-transduction channel with MEC-10^18^. These proteins interact with the paraoxonase-like MEC-6 and the cholesterol-binding stomatin-like MEC-2 protein which are required to sense gentle mechanical touch along the body wall^18^,^23^. TRP-4 is an N-type TRP channel which is a close homolog of NOMPC/TRPN1 in Drosophila^24^. TRP-4 is expressed in dopaminergic neurons such as CEP, PDE and DVA, and is involved in slowing the basal response, proprioception and in sensing ultrasound stimulus^17^,^19^,^25^. In the ASH neurons, loss of OSM-9 abolishes touch-evoked calcium elevations^1^. However, DEG-1, but not OSM-9, is required for the touch-receptor currents in ASH. This suggests that OSM-9 may act as a calcium modulator, but not as a touch receptor^16^. Here we found that OSM-9 was required for touch-induced calcium responses in the PHA/PHB neurons. Nevertheless, our data excludes a role of either DEG-1 or other ENaC in touch-induced calcium responses in PHA/PHB. Further efforts might be expended to identify which mechano-gated channel(s) function as the mechano-receptor(s) in these neurons. The answer to this question may shed new insights into the long-lasting attempt to identify the mechno-gated channels mediating hearing, touch-sensation and pain in mammals^23^,^26^,^27^.

A single type of sensory neuron responding to different kinds of stimuli represents an intriguing problem in neurology. Our data suggests that the combined approach of CNG signaling, TRP signaling, and neuropeptide signaling are responsible for encoding and discriminating between different kinds of stimuli in the PHA/PHB neurons. This observation may help us to uncover the other mechanisms of polymodal signaling such as nociception in more complex organisms.

## Materials and Methods

### Strains

*C. elegans* strains were maintained under standard conditions^28^. We generated a transgenic strain kanEx178 [P*ocr-2*::dsRed + P*ocr-2*::GCaMP5] to monitor intracellular activities in the PHA/PHB neurons. Mutant strains included: *osm-9(ky10)* kanEx178; *tax-4(ky11)* kanEx178; *deg-1(u38)* kanEx178; *unc-13(e51)* kanEx178; *unc-31(e928)* kanEx178.

### Calcium Imaging

A drop of bath solution containing a D2 adult worm was placed on a coverslip. Then the worm was glued to the pad with a cyanoacrylate-based glue (Gluture Topical Tissue Adhesive, Abbott Laboratories). The anus segment was exposed to chemical and mechanical stimuli. The calcium indicator GCaMP5 was used to measure the intracellular calcium signals. Imaging was acquired in an Olympus microscope (BX51WI) with a 60 × objective lens on an Andor DL-604M EMCCD camera. Data was collected using the Macro-manager software. GCaMP5 was excited by a Lambda XL light source and fluorescent signals were collected at a rate of 1 Hz. The average GCaMP5 signal from the first 3 s before stimulus was taken as F0, and ΔF/F0was calculated for each data point. The data was analyzed using Imaging J. The bath solution contained (in mM):145 NaCl,2.5 KCl, 1 MgCl_2_, 5 CaCl_2_,10 HEPES, 20 glucose. (325~335 mOsm, pH adjusted to 7.3 with NaOH).

### Mechanical Stimulation

Touch stimuli was delivered to the cell using a tip diameter of ~1 μm borosilicate glass capillary driven by a piezoelectric actuator (PI) mounted on a micromanipulator (Sutter). The needle was placed perpendicular to the worm’s body. In the “on” phase, the needle was moved toward the worm’s tail so that it could probe into the worm’s tail on the cilia and then held on the cilia for 500 ms. In the “OFF” phase the needle was returned to its original position.

### Statistical analysis

Data analysis was performed using Excel 2010 and Image J.Error bars were mean ± SEM. N represents the number of cells. P values were determined by Student’s t test. P < 0.05 was regarded as statistically significant.

## Additional Information

**How to cite this article**: Zou, W. *et al*. Polymodal Responses in *C. elegans* Phasmid Neurons Rely on Multiple Intracellular and Intercellular Signaling Pathways. *Sci. Rep.* **7**, 42295; doi: 10.1038/srep42295 (2017).

**Publisher's note:** Springer Nature remains neutral with regard to jurisdictional claims in published maps and institutional affiliations.
